# Crystal Structure of Pim1 Kinase in Complex with a Pyrido[4,3-*D*]Pyrimidine Derivative Suggests a Unique Binding Mode

**DOI:** 10.1371/journal.pone.0070358

**Published:** 2013-07-31

**Authors:** Sang Jae Lee, Byeong-Gu Han, Jea-Won Cho, Jang-Sik Choi, Jaekyoo Lee, Ho-Juhn Song, Jong Sung Koh, Byung Il Lee

**Affiliations:** 1 Biomolecular Function Research Branch, Research Institute, National Cancer Center, Goyang, Gyeonggi, Republic of Korea; 2 Oscotec Inc., Seongnam, Gyeonggi, Republic of Korea; 3 Genosco, Cambridge, Massachusetts, United States of America; 4 The Research Institute of Pharmaceutical Sciences, College of Pharmacy, Seoul National University, Seoul, Republic of Korea; Medical School of Hannover, United States of America

## Abstract

Human Pim1 kinase is a serine/threonine protein kinase that plays important biological roles in cell survival, apoptosis, proliferation, and differentiation. Moreover, Pim1 is up-regulated in various hematopoietic malignancies and solid tumors. Thus, Pim1 is an attractive target for cancer therapeutics, and there has been growing interest in developing small molecule inhibitors for Pim1. Here, we describe the crystal structure of Pim1 in complex with a newly developed pyrido[4,3-*d*]pyrimidine-derivative inhibitor (SKI-O-068). Our inhibitor exhibits a half maximum inhibitory concentration (IC_50_) of 123 (±14) nM and has an unusual binding mode in complex with Pim1 kinase. The interactions between SKI-O-068 and the Pim1 active site pocket residue are different from those of other scaffold inhibitor-bound structures. The binding mode analysis suggests that the SKI-O-068 inhibitor can be improved by introducing functional groups that facilitate direct interaction with Lys67, which aid in the design of an optimized inhibitor.

## Introduction

Human Pim1 kinase is a serine/threonine protein kinase that belongs to the calcium/calmodulin-regulated kinases (CAMK) group [1]. Pim1 can be induced by various cytokines, mitogens and hormones, primarily through JAK/STAT and NF-κB [2]–[4]. Pim1, Pim2 and Pim3 kinases are overexpressed in various hematological malignancies and solid tumors, and the overexpression is correlated with poor prognosis [5], [6]. Pim1 kinase contributes to tumorigenesis by stimulating cell cycle progression and inhibiting apoptosis through various target molecules, such as Cdc25A, Cdc25C, p21^cip1/waf1^, p27^Kip1^, NuMA, HP1β, dynein, dynactin, Bad, Ask1, and Pras40 [4]. Moreover, Pim1 and Pim2 are conferring drug-resistance in prostate cancers and lymphomas, respectively [7], [8].

In contrast to other protein kinases, Pim1 kinase is constitutively active, and its activity is primarily regulated through protein expression and stability rather than posttranslational modification, although regulation by phosphorylation has been reported [4]–[6], [9], . The most promising aspect of Pim1 is that the structural conformation of the hinge region at the ATP-binding pocket differs from that of other protein kinases, which may aid in the design of more specific and selective inhibitors [5]. Moreover, Pim1 knockout mice show a mild phenotype, which suggests that Pim1 inhibitors may have few side effects [6]. Thus, Pim1 is an attractive target for cancer therapeutics, and there has been growing interest in developing small-molecule inhibitors for Pim1 [4], [11].

Many inhibitor-bound Pim1 crystal structures are available in the Protein Data Bank [5]. These inhibitors can be classified into three major classes based on their binding mode [5], [12]. The first class of inhibitors interacts with the Pim1 (Glu121) hinge region, and they are ATP-mimetic inhibitors. However, the second class of inhibitors interacts with the active site lysine (Lys67) rather than the Glu121 in the hinge region, and they are non-ATP mimetic/ATP-competitive inhibitors [5]. A third binding mode has been observed for certain inhibitors, which have characteristics of both ATP-mimetic and non-ATP mimetic binding modes [12]. A phase I evaluation was initiated for SGI-1776, which inhibits all Pim kinases; however, the clinical trials were terminated early because of cardiac toxicity [7], [13]. Therefore, a new therapeutic strategy and discovery of new inhibitors is necessary for further inhibitor development.

We have focused on developing a Pim1 inhibitor and have performed an X-ray crystallographic study to gain structural insights for lead compound optimization. Here, we report a crystal structure for Pim1 in complex with a newly developed inhibitor (SKI-O-068). Our inhibitor (SKI-O-068) exhibits a 133 nM half maximum inhibitory concentration (IC_50_) and a unique binding mode with the Pim1 kinase. Interactions between SKI-O-068 and the Pim1 active site pocket residues are different from those of previously reported inhibitor-bound Pim1 structures. We suggest that the structural information from the Pim1-inhibitor complex will be helpful in structure-based lead compound inhibitor optimization.

## Materials and Methods

### Chemistry and Measurement of Kinase Activity

**Figure 1:**
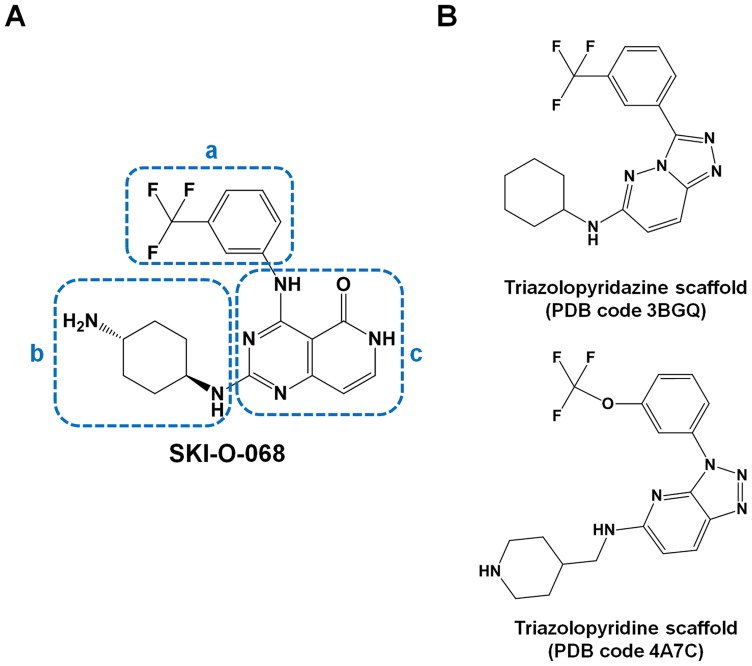
Structures for the ligands analyzed in this study. (A) Chemical structure of SKI-O-068. Three moieties on the ligand are indicated by blue-colored dashes [(a) the trifluoromethyl benzene moiety, (b) cyclohexanamine moiety and (c) pyrido[4,3-*d*]pyrimidin-5(6*H*)-*one*] moiety. (B) Two chemical scaffolds similar to SKI-O-068 (the triazolopyridazine scaffold from PDB code 3BGQ and triazolopyridine scaffold from PDB code 4A7C).

The inhibitor SKI-O-068 (IUPAC name: 2-(((1R,4R)-4-aminocyclohexyl)amino)-4-((3-(trifluoromethyl)phenyl)amino)pyrido[4,3-*d*]pyrimidin-5(6*H*)-one) was synthesized and supplied by Oscotec and Genosco (Fig. 1A). The compound was prepared from 2-(methylthio)-4-(3-(trifluoromoehyl)phenylamino) pyrido[4,3-*d*]pyrimidin-5(6*H*)-one and (1R,4R)-cyclohexane-1,4-diamine.

Biochemical kinase assays for Pim1, Syk, and Pyk2 kinase from Carna Biosciences Inc. were performed in accordance with the Perkin-Elmer TR-FRET protocol using suggested experimental materials, such as kinase and substrate. Briefly, the indicated SKI-O-068 concentrations ranged from 0.1 pM to 30 µM (final concentration in 2% DMSO) and incubation as performed with 148 µM ATP, 50 nM ULight-CREBtide peptide, and 332 pg/uL enzymes for 60 min. Following addition of a Europium-labeled anti-phospho-CREB antibody, the kinase reaction was terminated by EDTA. The Europium signal at 665 nm was detected using time-resolved fluorescence via Envision. The IC_50_ was calculated using nonlinear regression and GraphPad Prism 5. Staurosporine was used as a control for the experimental conditions.

### Protein Purification and Crystallization

An N-terminal truncated form (residues 29−313) of the human pim1 gene was amplified using PCR and cloned into a modified pET32 vector (Novagen) using NdeI and NotI restriction sites, respectively. The residue numbering in this work followed previous structure reports [10], [14]. The construct used herein included twelve extra residues (AAALEHHHHHHHH) at the carboxyl terminus of the recombinant proteins. The recombinant protein was overexpressed in *E*. *coli* Rosetta2(DE3)pLysS (Novagen) cells using Terrific-Broth culture medium. After growth to the mid-log phase at 37°C, protein expression was induced by 1 mM isopropyl 1-thio-β-D-galactopyranoside, and the cells were further incubated for 20 h at 22°C. The cells were lysed by sonication in lysis buffer (10 mM Tris-HCl at pH 7.4, 2.5 mM KCl, 100 mM NaCl, 1 mM phenylmethylsulfonyl fluoride, and 1 µM lysozyme) with 10% (v/v) glycerol. The crude lysate was centrifuged at ∼36,000 *g* for 60 min. The supernatant was applied to a Ni^2+^-NTA column (Qiagen) for affinity purification via the C-terminal octa-histidine tag. The eluent was pooled and concentrated. The protein was further purified by gel filtration on a Superdex 75 prep-grade column (GE Healthcare), which was previously equilibrated using 20 mM Tris-HCl buffer at pH 7.5 with 200 mM NaCl and 1 mM β-mercaptoethanol. The Pim1-containing fractions were concentrated to 9.2 mg/ml for crystallization using an Amicon Ultra-15 centrifugal filter unit (Millipore).

### Crystallization and Data Collection

To grow crystals of inhibitor-free and inhibitor-bound Pim1, we incubated the protein solution at 24°C for one hour after adding the inhibitor dissolved in dimethyl sulfoxide at a 5-fold molar excess. The crystals were grown using the sitting-drop vapor diffusion method at 4°C by mixing equal volumes (2 µl each) of the protein solution and reservoir solution that comprised 0.7 M sodium potassium tartrate and 0.1 M 2-(N-morpholino)ethanesulfonic acid (MES) buffer (pH 6.5). The crystals were grown to approximately 0.02 mm ×0.02 mm ×0.4 mm within a week.

X-ray diffraction data were collected on an ADSC Quantum 210 CCD detector (Area Detector Systems Corporation, Poway, CA, USA) under cryogenic conditions at the BL-6C experimental station in Pohang Light Source, Korea. For each image, the crystal was rotated 1°, and the raw data were processed and scaled using the program suit HKL2000 (Otwinowski, Z., and W. Minor. 1997. Methods Enzymol) [15]. The crystals belonged to the hexagonal space group P6_5_. Each asymmetric crystal unit comprised a single Pim1 monomer.

### Structure Determination and Refinement

The Pim1 structures were determined using molecular replacement and the program Molrep [16] by employing a Pim1 model (PDB code 1XQZ) [10] for searches. Five percent of the data were randomly used as a test set to calculate R_free_ [17]. The models were manually constructed using the program Coot [18] and refined with using the programs Phenix [19] and Refmac [20], which included bulk solvent correction. The inhibitor (SKI-O-068) and water molecules were assigned based on *mFo* – *DFc* maps calculated using the model phases. The models demonstrated excellent stereochemistry, which was evaluated using the program MolProbity [21]. Structural deviation was calculated using Superpose [22]. Table 1 summarizes the refinement statistics.

**Table 1:** Statistics from data collection and model refinement.

Data collection		
Data set	Native	Inhibitor-bound form
X-ray wavelength (Å)	1.2399	1.2399
Space group	P6_5_	P6_5_
Unit cell parameters (Å)	*a, b* = 97.62	*a, b* = 97.66
	*c* = 80.62	*c* = 81.11
Resolution range (Å)	50–2.5 (2.59–2.50)^a^	50–2.4 (2.49–2.40)^a^
Total/unique reflections	94,106/15,115	97,447/16,996
Completeness (%)	98.3 (99.5)^a^	99.4 (99.9)^a^
<*I*/*σ*(*I*)>	38.7 (4.4)^a^	24.3 (3.5)^a^
*R*_merge_ b	0.054 (0.463)^a^	0.066 (0.461)^a^
**Model refinement**		
*R*_work_/*R*_free_ c	0.221/0.249	0.191/0.227
No. of nonhydrogen atoms/average *B*-factor (Å^2^)		
Protein	2,227/47.2	2,279/38.2
Water oxygen	67/43.2	104/38.9
Inhibitor		30/45.6
R.m.s. deviations from ideal geometry		
Bond lengths (Å)/bond angles (°)	0.009/1.30	0.009/1.31
R.m.s. Z-scores^d^		
Bond lengths/bond angles	0.443/0.581	0.470/0.591
Ramachandran plot (%)		
Favored	93.4^e^	98.2^e^
Outliers	0.0^e^	0.0^e^
Rotamer outliers (%)	0.82^e^	1.20^e^

a Values in parentheses refer to the shell with the highest resolution.

b *R*_merge_ = Σ_h_ Σ_i_ | *I*(*h*)_i_ –<*I*(*h*)>|/Σ_h_ Σ_i_ *I*(*h*)_i_, where *I*(*h*) is the intensity for reflection *h*, Σ_h_ is the sum for all reflections, and Σ_i_ is the sum for i measurements of reflection *h*.

c *R* = Σ | |*F*_obs_| – |*F*_calc_| |/Σ |*F*_obs_|, where *R*_free_ is calculated for a randomly chosen 5% of reflections, which were not used for structure refinement, and *R*_work_ is calculated for the remaining reflections.

d Values generated using Refmac [20].

e Values generated using MolProbity [21].

### Protein Data Bank Accession Numbers

The atomic coordinates and structure factors for human Pim1 (the inhibitor-free form and inhibitor-bound form) are in the Protein Data Bank under accession codes 4JX3 and 4JX7, respectively.

## Results and Discussion

### Discovery of the Pim1 Kinase Inhibitor

In the development of a Pim1 inhibitor for anticancer drug development, we discovered SKI-O-068, which was an initial hit. For this discovery, one hundred compounds from in-house library were screened against human Pim1 kinase and initial hits were selected. In a biochemical enzyme assay, SKI-O-068 inhibited Pim1 with an IC_50_ value of 123 (±14) nM. This compound also inhibited Syk kinase with an IC_50_ of 53 (±10) nM and Pyk2 kinase with an IC_50_ of 55 (±10) nM, which suggests that SKI-O-068 is multi-potent kinase inhibitor. Because it was a potent inhibitor for Pim1, we determined its crystal structure for lead compound optimization.

### Structure Determination and Model Quality

**Figure 2:**
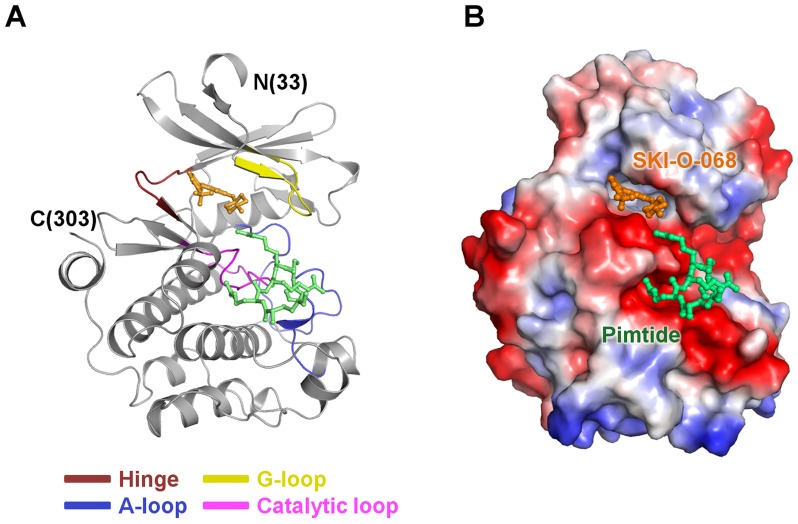
Overall structure for the complex of Pim1, SKI-O-068, and pimtide. (A) Ribbon diagram for the monomeric Pim1 structure. The hinge region, G-loop, A-loop and catalytic loop are colored raspberry, yellow, blue and pink, respectively. (B) Electrostatic potential surface diagram for the monomeric Pim1 structure. SKI-O-068 and pimtide are shown in orange and green, respectively. The inhibitor is in the active site, and pimtide is in the active-binding pocket vicinity. The structure figures and surface diagram were drawn using PyMOL (DeLano WL, 2002, The PyMOL Molecular Graphics System. DeLano Scientific, Palo Alto, California).

We determined two crystal structures in this study: those of inhibitor-free Pim1 and inhibitor (SKI-O-068)-bound Pim1 (Fig. 2). The inhibitor-free Pim1 structure was refined using 2.4 Å data to R_work_ and R_free_ values of 0.221 and 0.249, respectively. It included 274 amino acid residues in one monomer and 68 water molecules in the asymmetric unit. The refined model for inhibitor-bound Pim1 includes 274 residues in one monomer, one consensus substrate peptide (residues KRRRH), and 104 water molecules in the asymmetric unit. It produced R_work_ and R_free_ values at 0.191 and 0.227 for the 20–2.40 Å data, respectively. Eleven residues (Lys29–Glu32 and Leu307–Lys313) and twelve residues from the C-terminal fusion tag are disordered in both structures. The inhibitor-free Pim1 structure is highly similar to the inhibitor-bound Pim1 structure with a 0.263 Å root mean square (r.m.s.) deviation for 223 Cα atom pairs. The refinement statistics are summarized in Table 1.

### Binding Mode for Pimtide in the Substrate Binding Site

**Figure 3:**
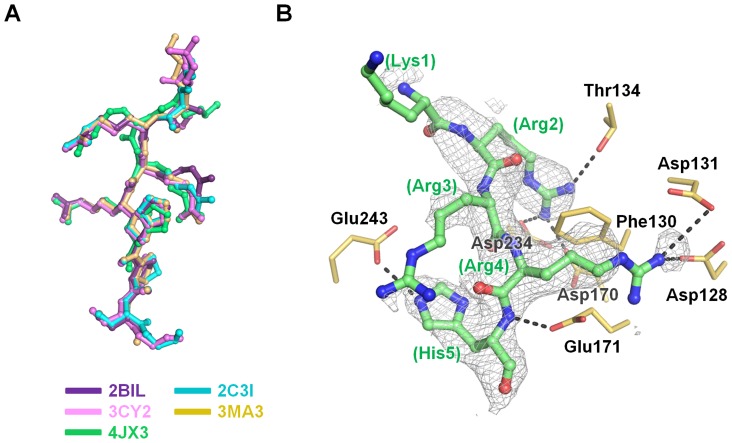
The pimtide binding mode at the substrate-binding pocket. (A) Superposition of pimtide molecules in pimtide-bound structures. PDB codes 2BIL (purple), 2C3I (cyan), 3CY2 (light pink) and 3MA3 (ivory) were used for structural comparison. (B) The pimtide binding mode (green). The *Fo* – *Fc* electron density map is contoured at 2.5 σ and colored in gray. The polar interactions are depicted using gray-colored dashes.

Although a synthetic peptide was not added during protein purification and crystallization, additional election density was observed at the substrate-binding site for the inhibitor-bound structure, which is not a water molecule. Various structural reports for Pim1 have demonstrated binding of a consensus substrate peptide (pimtide; ^1^ARKRRRHPSGPPTA^14^), and a portion of the pimtide sequence was fit to the electron density. Thus, we tentatively modeled five residues from pimtide (^3^KRRRH^7^) to the additional election density at the substrate-binding site (Fig. 2). In the inhibitor-bound Pim1 structure, pimtide yielded a structure identical to previously reported Pim1 structures (PDB codes 2BIL, 2BZK [23], 2C3I [24], 3CY2 [25], and 3MA3 [26]), wherein the pimtide was modeled into the substrate-binding pocket (Fig. 3A). Three residues (Arg^4^, Arg^6^, and His^7^) of five well-defined pimtide residues interact with Pim1. Arg^4^ interacts with Thr134, Asp170, and Asp234. Arg^6^ contacts Asp128, Asp131, the Phe130 carbonyl moiety, and Glu171. His^7^ forms interactions with Glu171 and Glu243 (Fig. 3B).

### Structural Analysis of Inhibitor-Bound Pim1

**Figure 4:**
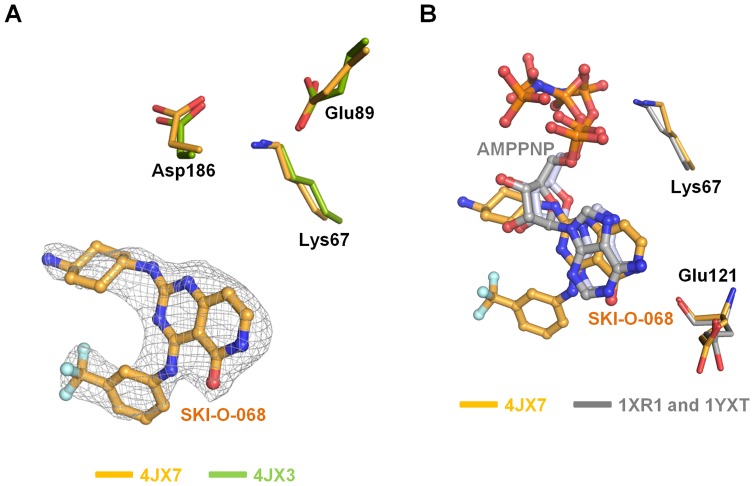
Comparison of SKI-O-068 and AMPPNP. (A) The *Fo* – *Fc* electron density map (in gray and contoured at 3.0 σ) and three key residues (Lys67, Glu89 and Asp186) are depicted. The SKI-O-068-bound and native residues are compared in yellow and green, respectively. (B) SKI-O-068 (orange) superposition with two AMPPNPs (1XR1 and 1YXT; gray). Both Lys67 and Glu121 are depicted.

Pim1 has a typical serine/threonine kinase fold comprising two domains [N-terminal domain (NTD), residues 33–120; C-terminal domain (CTD), residues 129–305], which are linked by a hinge region with a unique LERPXPX motif and the gatekeeper residue (Leu120). The ATP binding pocket is between the NTD and CTD, and it is surrounded by the hinge region, glycine-rich loop (G-loop, residues 46–54), and activation loop (A-loop, residues 191–202) [5] (Fig. 2A). For the inhibitor-bound structure, electron density was clearly observed at the ATP binding pocket and assigned as the SKI-O-068 inhibitor (Fig. 4A). The A-loop comprises the conserved DFG motif, and the A-loops for the SKI-O-068-bound and inhibitor-free Pim1 structure show an active “DFG-in” conformation, which is similar to other Pim1 structures. Hydrogen bonds between Lys67 and Glu89 facilitate a sustained active A-loop conformation [5]. Moreover, Lys67 is critical to Pim1 catalytic activity and in ATP-bound structures has been shown to form multiple hydrogen bonds with Asp186 (Asp residue of the 'DFG' motif), a magnesium ion, and an ATP phosphate group [10], [27]. The structural conformation and hydrogen bond networks among Lys67, Glu89, and Asp186 are well conserved in our inhibitor-bound and inhibitor-free structures (Fig. 4A) [10], [14], [28]. Previous reports have shown that Pim1 adopts a constitutively active conformation regardless of the phosphorylation state, which suggests that its activity is regulated through expression and protein stability [4], [10]. Two strategies have been suggested for kinase inhibition: inhibition of the active kinase conformation and a stabilized inactive kinase conformation [29]. Therefore, the constitutively active Pim1 conformation offers a great advantage because only one kinase structure conformation must be considered for inhibitor design. A conformational change in the G-loop, including a Phe49 flip, has been reported in certain cases [10], [14], [26], [30]. A conformational change in the G-loop was not observed in the SKI-O-068-bound Pim1 structure.

In two AMPPNP-bound structures (PDB codes 1XR1 and 1YXT), the phosphate groups, and adenosine moieties contact Lys67 and Glu121, respectively [10], [14]. Superimposition of the SKI-O-068-bound structure and two AMPPNP-bound structures shows that the SKI-O-068 cyclohexanamine moiety is positioned similarly to the AMPPNP ribose moiety, and the SKI-O-068 pyrido[4,3-*d*]pyrimidin-5(6*H*)-*one* moiety is superimposed with the AMPPNP adenosine moiety (Fig. 4B).

**Figure 5:**
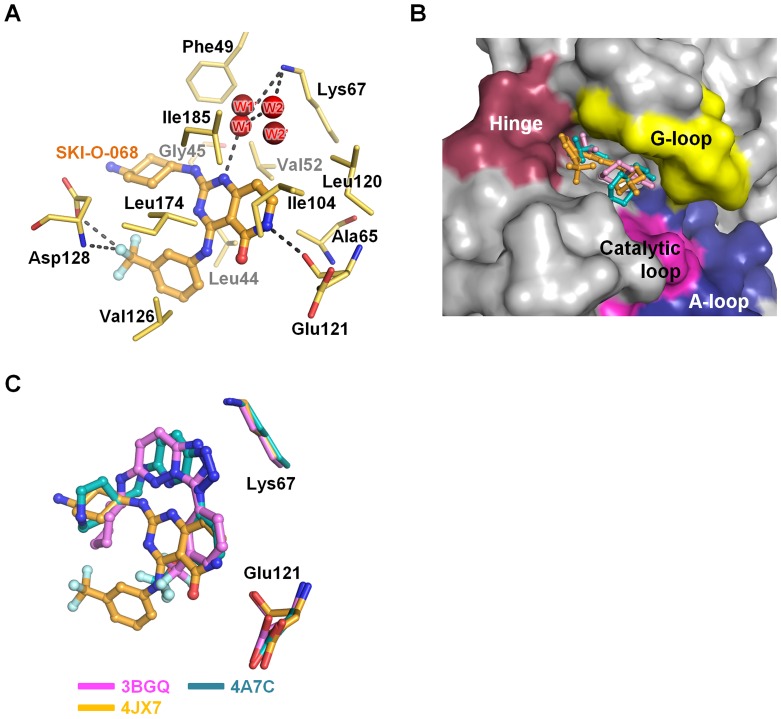
The SKI-O-068 binding mode in the Pim1 active site. (A) SKI-O-068 intermolecular interactions. The two water molecules (W1 and W2) are from the SKI-O-068-bound Pim1 structure. The additional water molecules (W1’ and W2’) are from the native Pim1 structure. The water molecules are depicted as spheres in red. The polar interactions are depicted using gray dashes. (B) The Pim1 surface diagram depicting the active site pocket. Three inhibitors from Figure 1 are superimposed. The molecular surface is depicted in raspberry (hinge), yellow (G-loop), blue (A-loop) and pink (catalytic loop). (C) Detailed view of three inhibitors and two residues (Lys67 and Glu121).

The structure for Pim1 with the inhibitor SKI-O-068 was compared with the inhibitor-free structure. In the SKI-O-068-bound structure, the trifluoromethyl phenyl moiety of SKI-O-068 was recognized by Leu44 (NTD), Val126 (Hinge region), Asp128 (CTD), and Leu174 (CTD) (Fig. 5A). The SKI-O-068 cyclohexanamine moiety interacts hydrophobically with Gly45 (NTD), Leu174 (CTD), and Ile185 (CTD) (Fig. 5A). The SKI-O-068 pyrido[4,3-*d*]pyrimidin-5(6*H*)-*one* moiety forms a direct hydrogen bond with the Glu121 carbonyl moiety (hinge region), and it forms hydrophobic interactions with Val52 (NTD, G-loop), Ala65 (NTD), Ile104 (NTD), Leu120 (NTD), Leu174 (CTD), and Ile185 (CTD) (Fig. 5A). The hydrophobic interactions described above determine the affinity of inhibitors for the ATP-binding pocket [12].

Pim1 inhibitors are classified into three groups in accordance with the binding mode. These groups include ATP-mimetic inhibitors that interact with Glu121 in the hinge region, non-ATP-mimetic inhibitors that interact with Lys67 and a third group of inhibitors that simultaneously interact with both Lys67 and Glu121 [12]. ATP-mimetic inhibitors demonstrate broad inhibitory ability and include staurosporine [31] as well as LY333531 [24]. Non-ATP-mimetic/ATP-competitive inhibitors include LY294002 [31] and (E)-3-{3-[6-(4-aminocyclohexylamino)-pyrazin-2-yl]phenyl}acrylicacid [28]. The third group of inhibitors include the flavonol quercetagetin and 4-(4-hydroxy-3-methylphenyl)-6-phenylpyrimidin-2(1H)-one compound [12], [32], [33]. Additionally, certain inhibitors are borderline inhibitors that interact with Lys67 and form weak hydrogen bonds with Glu121. This group includes imidazo[1,2-*b*]pyridazine [24], the substituted pyridine [30], and triazolo[4,3-*b*]pyridazine [34]. The SKI-O-068 chemical scaffold resembles the triazolopyridazine (PDB code 3BGQ; Ki ∼11 nM) [35] and triazolopyridine scaffolds (PDB code 4A7C; IC_50_ ∼6 nM) [36] in the borderline group (Fig. 1B). Although these inhibitors occupy the same binding pocket (Fig. 5B), the SKI-O-068 binding mode is distinct from that of triazolopyridazine and triazolopyridine derivative inhibitors. The triazolopyridazine and triazolopyridine scaffolds simultaneously interact with both Lys67 (strong) and Glu121 (weak) (Fig. 5C). However, SKI-O-068 demonstrates characteristics of ATP-mimetic inhibitors. It directly interacts with Glu121. Additionally, it indirectly interacts with Lys67 through water molecules (W1 or W2) (Fig. 5A). Moreover, SKI-O-068 directly interacts with Asp128 (Fig. 5A), which is similar to certain ATP-mimetic inhibitor binding modes [12]. From the binding mode analysis, the SKI-O-068 inhibitor can be improved by introducing functional groups that directly interact with Lys67. Furthermore, such improved inhibitors should demonstrate strong binding affinity for Pim1.

In conclusion, we discovered a potential Pim1 inhibitor, SKI-O-068, and we produced crystal structures for both the inhibitor-bound and inhibitor-free forms. In the crystal structure, the SKI-O-068 inhibitor-bound Pim1 was distinct from structurally similar scaffold inhibitors. Structural information and a binding mode analysis for SKI-O-068 will facilitate improved rational drug design. The *in vitro* ADME/TOX testing on SKI-O-068 as well as rational design to improve inhibitor is now in progress. Further studies on cytotoxicity and pharmacokinetics/pharmacodynamics should be completed for further development of this drug as an anticancer agent.
