# Correction: Crystal Structure of Pim1 Kinase in Complex with a Pyrido[4,3-*D*]Pyrimidine Derivative Suggests a Unique Binding Mode

**DOI:** 10.1371/journal.pone.0190843

**Published:** 2018-01-02

**Authors:** Sang Jae Lee, Byeong-Gu Han, Jea-Won Cho, Jang-Sik Choi, Jaekyoo Lee, Ho-Juhn Song, Jong Sung Koh, Byung Il Lee

There is an error in the first sentence of the “Protein Purification and Crystallization” section of the Materials and Methods. The correct sentence is: An N-terminal truncated form (residues 29−313) of the human pim1 gene was amplified using PCR and cloned into a modified pET28 vector (Novagen) using NdeI and NotI restriction sites, respectively.
